# Assessing the Genetic Risk for Alcohol Use Disorders

**DOI:** 10.35946/arcr.v34.3.01

**Published:** 2012

**Authors:** Tatiana Foroud, Tamara J. Phillips

**Affiliations:** **Tatiana Foroud, Ph.D.,***is a professor in the Department of Medical and Molecular Genetics, Indiana University School of Medicine, Indianapolis, Indiana.*; **Tamara J. Phillips, Ph.D.,***is a professor in the Department of Behavioral Neuroscience, Oregon Health & Science University, and a senior research career scientist at the Portland Veterans Affairs Medical Center, Portland, Oregon.*

**Keywords:** Alcohol dependence, alcoholism, genetics, genetic basis of alcoholism, genetic mapping, gene interactions, genetic technology, gene knockout technology, human studies, animal models

## Abstract

The past two decades have witnessed a revolution in the field of genetics which has led to a rapid evolution in the tools and techniques available for mapping genes that contribute to genetically complex disorders such as alcohol dependence. Research in humans and in animal models of human disease has provided important new information. Among the most commonly applied approaches used in human studies are family studies, case–control studies, and genome-wide association studies. Animal models have been aimed at identifying genetic regions or individual genes involved in different aspects of alcoholism, using such approaches as quantitative trait locus analysis, genome sequencing, knockout animals, and other sophisticated molecular genetic techniques. All of these approaches have led to the identification of several genes that seem to influence the risk for alcohol dependence, which are being further analyzed. Newer studies, however, also are attempting to look at the genetic basis of alcoholism at the level of the entire genome, moving beyond the study of individual genes toward analyses of gene interactions and gene networks in the development of this devastating disease.

According to the World Health Organization (http://www.who.int/substance_abuse/facts/alcohol/en/index.html), each year alcohol causes 2.5 million (3.8 percent of total) deaths and 69.4 million (4.5 percent of total) disability-adjusted life-years (DALYs) lost to disease worldwide. Alcohol dependence (alcoholism) also is a major health problem in the United States, affecting 4 to 5 percent of the population at any given time (Grant et al. 2004); its lifetime prevalence is 12.5 percent (Hasin et al. 2007). Initially, it was unclear whether environmental factors, genetic factors, or both contributed to the risk for alcohol dependence. Early studies clearly demonstrated that genes have a role in the risk for alcohol dependence; however, it also is clear that a substantial portion of the risk for alcoholism is not genetically determined and may result from other factors, such as the environment in which a person is raised or peer influences. In addition, gene–environment interactions exist that modify alcoholism risk (for more information, see the accompanying article by Dick and Kendler, pp. 318–324).

Ever since it has become clear that genetic factors influence the risk for alcoholism, researchers have sought to identify the genes involved. However, the complex nature of alcohol dependence and related disorders has slowed progress in identifying these genes. Thus, existing data suggest that each individual genetic element has only a small influence and that it will be necessary to identify the relevant gene networks to gain a greater understanding of the contribution of genetics to alcohol abuse and dependence (for more information on genetic and molecular networks of risk, see the article by Wolen and Miles, pp. 306–317).

Historically, two major approaches have been used to determine the magnitude of the overall genetic contribution to alcohol dependence in specific populations. The first approach was to compare the similarity (i.e., concordance) for alcohol dependence among identical (i.e., monozygotic) and fraternal (i.e., dizygotic) twins—that is, these studies assessed whether if one twin had alcohol dependence the other twin did so as well. If the risk for alcoholism, at least in part, results from genetic factors, one would expect monozygotic twins, who have identical genomes, to have a higher concordance rate for alcohol dependence than dizygotic twins, who on average only share one-half of their genomes. Studies indeed have shown higher concordance rates among monozygotic twins, confirming the presence of a genetic component in the risk for alcoholism. The second approach involved family studies to estimate the overall similarity among family members sharing differing proportions of their genome (e.g., comparing sons with fathers or grandfathers). Together, these family and twin studies provided convergent evidence that genetic factors account for 50 to 60 percent of the total variance in the risk for alcohol dependence (Heath et al. 1997; McGue 1999).

On the basis of these findings, the next step was to identify specific genes that could influence the risk for alcoholism. Over the past three decades, new developments have made it possible to search for specific genes that influence the risk for alcohol dependence, both in human populations and in animal models. This article summarizes some of these approaches used in human populations and in studies of animal models. It also describes newer approaches aimed at analyzing the genetic basis of alcoholism at the level of the entire genome, thus moving beyond analyses of the roles of individual genes in the development of this devastating disease.

## Identifying Genes Contributing to the Risk for Alcoholism

### Approaches in Human Populations

In human studies, several strategies have been used to search for the genes that influence complex traits such as alcohol dependence, which are influenced by multiple genes with smaller effects rather than by one or more genes with larger effect sizes (Edenberg and Foroud 2006; also see the article by Agrawal and Bierut, pp. 274–282). One approach, often termed linkage analysis, involves studying families with multiple members who have alcohol dependence. This approach is based on the hypothesis that genes might have a greater effect in these families than in families with only a single alcoholic member. To perform this type of study, researchers recruited hundreds of families having two or more alcoholic members (see figure 1A) and analyzed DNA samples from both alcoholic and nonalcoholic family members at approximately 400 different positions within the human genome for sequence differences. The data then were examined to determine whether alcohol-dependent individuals within families shared common gene variants (i.e., alleles). Finally, the investigators reviewed the data across all families in a study to determine whether individuals with alcoholism seemed to have inherited particular parts of the genome. Those portions of the genome that seem to be shared are called quantitative trait loci (QTLs) and are hypothesized to include genes that contribute to the risk for alcoholism. The QTLs can be quite large, often covering 10 or 20 million base pairs that may include hundreds or even thousands of genes, of which the right one or ones (because more than one in the region could contribute) would need to be identified. Although this approach was quite challenging, investigators were able to locate several genome regions that are thought to include genes that contribute to the risk for alcohol dependence. However, conclusively identifying the relevant gene(s) from the many within each large region has proven to be more difficult than anticipated.

The second approach, called a case–control study, often has been used to examine the role of a single gene in complex disorders such as alcoholism. This strategy involves comparing two groups of individuals: people with alcohol dependence and control subjects who are not alcoholic, without regard to the participants’ family histories (see figure 1B). In this type of study, investigators analyze the distribution of sequence variants within or near a gene suspected to be involved in alcoholism in the two groups, using statistical methods to compare the frequencies either of different alleles or of the resulting genotypes between the two groups. If a certain allele contributes to the risk for alcohol dependence, one would expect the allele and/or genotype to occur more frequently among the alcohol-dependent case subjects than among the nonalcoholic control subjects. Case–control studies often have been performed on small numbers of alcoholics and control subjects, limiting their statistical power. Moreover, many results from these studies could not be replicated, although this inability may be caused by population differences in genetic risk. The most robust result to emerge from these studies was the demonstration that the genes involved in alcohol metabolism—that is, genes encoding the alcohol dehydrogenase and aldehyde dehydrogenase enzymes—play important roles in the risk for alcoholism (for more information, see the article by Hurley and Edenberg, pp. 339–344). In addition, such analyses have implicated several gene pathways that encode brain-signaling molecules (i.e., neurotransmitters) and the molecules that mediate the actions of opioids (i.e., opioid receptors) (for more information, see the article by Borghese and Harris, pp. 345–354), as well as genes in the neuroendocrine and neuroimmune system (see the article by Crews, pp. 355–361) and genes regulating circadian rhythms (see the article by Sarkar, pp. 362–366).

With the rapid advances in molecular genetic technology, it now is possible to test the entire genome rather than focus on individual genes suspected to play a role (i.e., candidate genes) or use genetic variants spaced at wide intervals throughout the genome. Although these new approaches do not test all 3 billion nucleotides that make up the human DNA sequence, they can test a few thousand (or in some cases a million or more) different positions within the genome (Stranger et al. 2010). This type of study, which is called a genome-wide association study (GWASs) (Manolio 2010), allows a comprehensive test of association across the genome, often while comparing case and control subjects. GWASs have been used for many different diseases, with varying success. Several studies have now applied this approach to begin to tackle the genetics of alcohol dependence (see the article by Edenberg, pp. 336–338). However, the statistical power of GWASs is a significant hurdle. Thus, very large samples are needed because most genetic variants only have small effects, and many tests need to be performed when analyzing hundreds of thousands or a million of the genetic variants known as single nucleotide polymorphisms (SNPs). In addition, the frequency of the influential alleles in a population has an impact on the sample size that is needed to detect their influence. Furthermore, it is likely that the role that a specific allele plays in the risk for alcoholism may differ among individuals, even if they all seem to have the same disorder. This can be thought of as akin to differences among patients in response to different blood pressure medications: Although the patients all have high blood pressure, the genetic makeup may determine which medication will be most effective for a given patient.

### Approaches in Animals

Animal models of traits related to human alcohol use disorders can provide pertinent information about the human condition. The usefulness of this information depends on the validity of the animal model, and there is great interest in the level of consilience between the human and laboratory animal findings (for more information, see the article by Barkley-Levenson and Crabbe, pp. 325–335).

As in human studies, approaches aimed at identifying QTLs have been used in animal studies of alcohol-related traits, such as alcohol consumption and sensitivity to alcohol; however, the nature of the genetic models used varies somewhat from that used in human studies. Most of the data have come from studies performed in mice, although rats have been used as well, and a recent study has compared results for rats and humans (Tabakoff et al. 2009). In one commonly used strategy, two or more strains of mice that are known to differ with regard to the alcohol-related trait under investigation are mated to each other and their offspring (called the F1 generation) then are interbred to create a population (the F2 generation) in which the individual animals possess genes from each of the originating strains in different proportions. The F2 animals then are scored for the trait studied (e.g., amount of alcohol ingested), and samples of their DNA are analyzed (i.e., genotyped) to identify genetic differences that correspond to differences in the alcohol trait. Technological advances have reduced the labor and cost associated with genotyping, making it possible to handle larger numbers of samples and to reduce genotyping intervals. Then, QTL analyses are performed largely as described above for the human studies.

This approach has identified many regions that contain QTLs; however, determining which gene(s) in the region has a significant impact has been difficult and requires the use of additional methods. One such method is finer mapping using animal populations generated by applying other breeding strategies (for reviews of such strategies, see Milner and Buck 2010; Palmer and Phillips 2002). This approach has identified several genes for which strong evidence supports their role in alcohol-related traits (for more information, see the article by Buck et al., pp. 367–374). Currently, second-generation DNA-sequencing technologies, often called next-generation sequencing, are enhancing both sequencing efficiency and the detection of genetic differences (Metzker 2010), and third-generation sequencing is emerging (Schadt et al. 2010).

Perhaps the most popular strategy for examining the influence of single candidate genes has been the use of single-gene mutant, or knockout, mice. For this approach, genetic engineering is used to generate a mutant gene that no longer can express the protein it normally produces, and that nonfunctional (knockout) gene is inserted into the genome of test animals. Mice possessing the mutated gene then are compared with nonmutant mice for differences in alcohol-related traits. Between 1996 and 2006, this approach was used to study approximately 100 genes for their effects on alcohol-related traits (Crabbe et al. 2006), and more genes have been studied since. It is important to realize, however, that although a difference identified between knockout and normal mice provides evidence for a role of the gene studied, this cannot be considered definitive because many interpretational issues are associated with this genetic tool. Other, more refined, gene-manipulation strategies that do not entirely eliminate the activity of a gene can provide additional evidence for the influence of a given gene. These strategies include approaches such as conditional inactivation or rescue, in which gene activity is reduced or eliminated only under certain conditions that can be controlled by the researcher (e.g., Choi et al. 2002) and RNA interference, in which reduced gene expression occurs only in a small region of the brain (e.g., Lesscher et al. 2009; Rewal et al. 2009).

Animal models also can be useful in research directed toward understanding the health consequences of alcohol consumption, including consequences in different organs. Much of this research is aimed at exploring how alcohol affects the brain and how the brain influences different individual responses to alcohol that may contribute to the development of alcohol dependence. One strategy that can be used in studies of both the brain and other tissues is called whole genome expression profiling, which examines the activity (i.e., expression) of thousands of genes located throughout the genome (Rockman and Kruglyak 2006). Particularly in studies of the brain, however, this approach can be applied more readily to animals than to humans, because a sample of the tissue to be studied is needed. This tissue sample is used to extract a type of genetic material called messenger RNA (mRNA) that is generated during the process of gene expression. The mRNA then is added to a microarray—that is, a membrane or slide on which known gene sequences have been placed that can interact with complementary RNA sequences from the brain sample. The amount of mRNA interacting with the gene sequences on the microarray then can be measured, giving an indication of the level of gene expression for each gene studied. A more recent modification of this method called RNA-seq (because it involves determination of the mRNA building blocks, or RNA sequence) has allowed researchers to obtain even more detailed information on gene expression (Ozsolak and Milos 2011).

Whole-genome expression profiling can be used in different ways. For example, studying gene-expression differences between animals that are sensitive or resistant to a given alcohol effect can provide evidence for genes that influence sensitivity to that alcohol effect. Comparisons between alcohol-exposed and non–alcohol-exposed animals provide information about alcohol’s interactions with genes and gene expression.

Gene-expression studies also have been performed with human samples, using postmortem brain tissue from alcohol-dependent individuals and nondependent control subjects (e.g., Kryger and Wilce 2010). However, factors such as quality of the sample and incomplete history of the subject from which the tissue was obtained complicate interpretation. In animals, these factors can be better controlled. Finally, in addition to whole genomes being examined for expression differences using RNA, DNA microarrays have been developed for global examination of genomic variation (Gresham et al. 2008).

## Moving Beyond Studying One Gene at a Time

Using the approaches described here, researchers now have identified some genes that are thought to influence the risk for alcoholism in humans or to contribute to alcohol-related traits in animal models. However, it is clear that the genetics of alcoholism and alcohol-related traits are complex and will include not only the effect of individual genes but also the interaction of genes with each other, a phenomenon termed epistasis. Identification of these interactions is only just beginning and is complicated by the fact that such analyses often require large numbers of subjects or animals. However, this area likely will garner much research interest in the future.

For now, the more immediate goal is to use the information currently available about the genes important in alcohol dependence in an integrated way to identify how these genes might work together and to help researchers identify additional genes that may be involved (for more information, see the article by Buck et al., pp. 367–374). One approach in this direction has been to study the genes already identified and determine whether they might work together in known biological pathways or networks. Such methods initially were used with gene-expression data, but newer statistical methods now permit pathway analysis also to be applied to GWASs data (Holmans 2010). When evidence for a coordinated network of interactions is identified, other genes known from previous research to be members of that network immediately can be considered as candidate genes to be tested for their potential role in alcohol-related traits.

Researchers also are developing and testing more complex models. For example, an approach called genetical genomics simultaneously takes into consideration both the gene-expression and gene-sequence (QTL) information to narrow the set of possibly involved genes down to a smaller set based on convergent results (Farris et al. 2010). More recently, it also has become clear that some inherited characteristics cannot be attributed to specific gene sequences but must be related to epigenetic effects—heritable modifications that are not based on differences in DNA sequence. The mechanisms underlying these epigenetic effects are being carefully scrutinized (for more information, see the article by Starkman et al., pp. 293–305).

It is worth noting that all these network studies have become possible because of a growing understanding of the mouse and human genomes. In the coming years, genetic discoveries likely will grow along with further technological advances. And we can hope that all of these developments will someday allow researchers and clinicians to identify those individuals who are at greatest risk for developing alcoholism. A related significant goal of current alcohol-related genetic research is to identify interventions that might decrease the risk for this devastating disease in those most likely to be affected. Finally, researchers are striving to better understand the genetic basis of differences in the effects of alcohol between people who are susceptible to alcoholism and those who are resistant to it, so that better treatment medications can be developed.

## Figures and Tables

**Figure:**
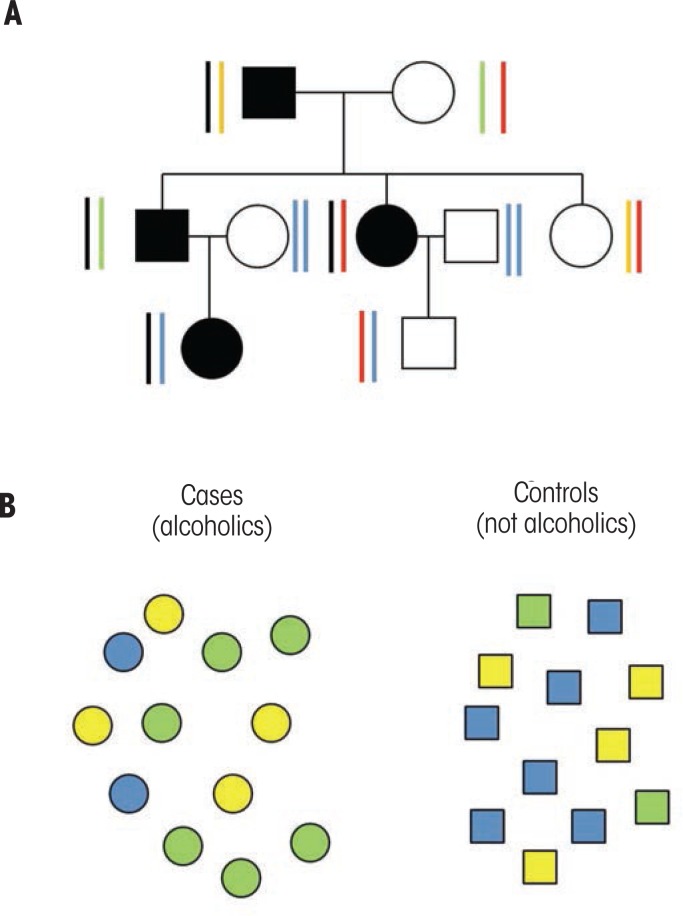
Strategies to identify genes contributing to alcohol dependence. **A)** Family study. In this figure, the squares represent males; the circles, females. The individuals with lines connecting their symbols produced children together and the lines down from that pair of individuals depict their offspring. Fully shaded symbols indicate individuals in the family who are alcohol dependent. The bars beside each symbol represent a region in the genome. Each individual has two copies of this region (one inherited from their mother and one from their father). The black bar carries a version of the gene (i.e., an allele) with a variation in its sequence that increases the risk of alcoholism. Notice that in this family, all four alcoholic individuals carry one copy of the allele that increases the alcoholism risk. The individuals who are not alcohol dependent do not carry this allele. If this pattern is repeated across many families, then there is likely to be a gene that influences the risk for alcoholism in this part of the genome. **B)** Case–control study. The three colors represent the three possible genetic makeups (i.e., genotypes) at the marker. The cases have more individuals with the green genotype and fewer with the blue genotype, whereas the controls have more individuals with the blue genotype and fewer with the green, suggesting that the green genotype is associated with an increased risk for alcohol dependence.
